# Nucleotide-Binding Oligomerization Domain 2 Contributes to Limiting Growth of *Mycobacterium abscessus* in the Lung of Mice by Regulating Cytokines and Nitric Oxide Production

**DOI:** 10.3389/fimmu.2017.01477

**Published:** 2017-11-06

**Authors:** Jun-Young Lee, Moo-Seung Lee, Dong-Jae Kim, Soo-Jin Yang, Sang-Jin Lee, Eui-Jeong Noh, Sung Jae Shin, Jong-Hwan Park

**Affiliations:** ^1^Laboratory Animal Medicine, College of Veterinary Medicine, Chonnam National University, Gwangju, South Korea; ^2^Infectious Disease Research Center, Korea Research Institute of Bioscience and Biotechnology, Daejeon, South Korea; ^3^Laboratory Animal Resource Center, Daegu Gyeongbuk Institute of Science & Technology (DGIST), Daegu, South Korea; ^4^School of Bioresources and Bioscience, Chung-Ang University, Anseong, South Korea; ^5^Department of Obstetrics and Gynecology, College of Medicine, Konyang University, Daejeon, South Korea; ^6^Department of Microbiology, Institute for Immunology and Immunological Diseases, Brain Korea 21 PLUS Project for Medical Science, Yonsei University College of Medicine, Seoul, South Korea

**Keywords:** nucleotide-binding oligomerization domain 2, muramyl dipeptide, *Mycobacterium abscessus*, macrophages, mitogen-activated protein kinases, nitric oxide

## Abstract

*Mycobacterium abscessus* is a prominent cause of pulmonary infection in immunosuppressed patients and those with cystic fibrosis. Nucleotide-binding oligomerization domain (NOD) 2 is a cytosolic receptor which senses a bacterial peptidoglycan component, muramyl dipeptide (MDP). Although nucleotide-binding oligomerization domain 2 (NOD2) contributes to protect host against various microbial infections, it is still unclear whether NOD2 is essential to regulate host immune responses against *M. abscessus* infection. In this study, we sought to clarify the role of NOD2 and the underlying mechanism in host defense against *M. abscessus* infection. Mice were infected intranasally with *M. abscessus* and sacrificed at indicated time points. Bacterial survival, cytokines production, and pathology in the lungs were determined. Bone marrow-derived macrophages were used to clarify cellular mechanism of NOD2-mediated immune response. Bacterial clearance was impaired, and pathology was more severe in the lungs of NOD2-deficient mice compared with the wild-type mice. In macrophages, NOD2-mediated activation of p38 and JNK were required for production of proinflammatory cytokines and nitric oxide (NO) and expression of iNOS in response to *M. abscessus*. NO was critical for limiting intracellular growth of the pathogen. Intranasal administration of MDP reduced *in vivo* bacterial replication and thus improved lung pathology in *M. abscessus*-infected mice. This study offers important new insights into the potential roles of the NOD2 in initiating and potentiating innate immune response against *M. abscessus* pulmonary infection.

## Introduction

*Mycobacterium abscessus* is a rapidly growing mycobacterium first isolated from a knee abscess in 1952 (1). It causes a variety of infections in humans, including soft tissue, skin, lung, and bone disease (2). Recently, its infection has been prominently recognized in patients with cystic fibrosis and other chronic pulmonary diseases (3–5). This pathogen has also become strongly resistant to most classical anti-tuberculous drugs and antibiotics, which thereby limits therapeutic options and often leads to a poor prognosis (6).

Nucleotide-binding oligomerization domain 2 (NOD2) is a member of cytosolic nucleotide-binding oligomerization domain (NOD)-like receptors family (7). Upon detecting muramyl dipeptide (MDP), a bacterial peptidoglycan component, NOD2 recruits receptor-interacting protein serine/threonine protein kinase 2 (RIP2/RICK/RIPK2) through the CARD–CARD interaction (8). Subsequently, RIP2 mediates recruitment and activation of TGFβ-activated kinase 1, which is required for activation of the IKK complex and mitogen-activated protein kinases (MAPKs) (9). Finally, this activation cascade leads to the expression of proinflammatory cytokines (9).

Previous studies have shown that NOD2 pathway contributes to the host innate immune response against pulmonary bacterial infections and that its role depends upon the species of bacterial pathogens (10–13). NOD2 is necessary for optimal phagocytosis of *Streptococcus pneumoniae* by alveolar macrophages and neutrophils *in vitro* (13). In addition, NOD2 cooperates with TLR2 to reduce the bacterial replication in the upper respiratory tract, although deficiency of NOD2 alone does not influence bacterial replication, dissemination, or lung pathology in mice infected with *S. pneumoniae* (12, 13). Moreover, NOD1/NOD2-RIP2 signaling pathways improved host immune responses against *Chlamydophila pneumoniae* infection, resulting in enhanced bacterial clearance in the lungs and longer survival time in the infected mice (10). In contrast, in a pulmonary infection model of *Legionella pneumophila*, NOD2 was dispensable for bacterial clearance in the lungs, although it was involved in the recognition of heat killed *L. pneumophila* (11).

The role of NOD2 in mycobacterial infections has been primarily studied in the *Mycobacterium tuberculosis* infection model, wherein NOD2 activation by MDP limited intracellular growth of *M. tuberculosis* within alveolar macrophages (14). NOD2 deficiency resulted in both increased bacterial loads in lung tissue and enhanced mortality during the chronic phase of infection, although the lung pathology during the early stage of infection was reduced (15). A recent study revealed that TLR2 is critical for initiating the protective Th1-type immune response against *M. abscessus*, but the exact role of NOD2 in initiating and propagating the host innate immune response against *M. abscessus* still remains unknown (16). To address these limitations in the present study, both the *in vitro* and *in vivo* roles of NOD2 were assessed in the context of host innate immunity against *M. abscessus* infection.

## Materials and Methods

### Mice

Wild-type (WT), NOD2, and iNOS-deficient mice on a C57BL/6 background were purchased from The Jackson Laboratory (Bar Harbor, ME, USA). The animal studies were conducted under protocols approved by the Institutional Animal Care and the Use Committee of Chonnam National University (Approval No. CNU IACUC-YB-2015-32).

### Bacterial Culture and Infection Experiments

Culture and single-cell suspensions of the isogenic rough variant of *M. abscessus* ATCC19977^T^ (Manassas, VA, USA) were performed as previously described (16). Briefly, the bacterium was cultivated in 7H9 broth supplemented with 0.5% glycerol, 10% oleic acid, albumin, dextrose, and catalase (OADC; BD Biosciences, San Jose, CA, USA) for 7–10 days. The seed lots were kept in small aliquots at −80°C until use. Mice were intranasally (i.n.) inoculated with 1.5 × 10^7^ CFU of bacteria. At 5, 10, and 20 days post infection (dpi), mice were sacrificed and the lung tissues were collected aseptically. For the *in vivo* MDP treatment, mice were i.n. dosed with *N*-glycolyl-MDP (150 μg/animal; Invivogen, San Diego, CA, USA), which exists in Mycobacteria (17).

### Lung Tissue Preparation and Bacterial Count

The right lobes of the lungs were weighed and homogenized with phosphate buffered saline (PBS). Half of the lysate was diluted with sterile PBS, plated onto 7H10 agar plates, and incubated at 37°C for 4 days. The colonies were counted and the number of bacteria (measured as CFU/g lung tissue) was calculated. The other half of the lysate was centrifuged and the supernatant was stored in a deep freezer for cytokine measurement.

### Histopathological Examination

The left lobes of the lungs were fixed in 10% neutral formalin for 24 h, followed by tissue processing and paraffin embedding. The paraffin blocks were sectioned at 2 µm, stained with hematoxylin and eosin, and examined under microscopy. The lung pathology was evaluated blindly by an expert in a field of laboratory animal pathology. with an arbitrary scoring index ranging 0–5, which was determined by the degree of inflammatory cell infiltration and the extent of the lesion area (0, normal; 1, mild; 2, mild to moderate; 3, moderate; 4, moderate to severe; 5, severe).

### Preparation and Infection of Murine Macrophages

Bone marrow-derived macrophages (BMDMs) were prepared as previously described (18). Briefly, bone marrow was extracted from femur and tibia of mice and BMDMs were cultured in Iscove’s modified Dulbecco’s medium (Gibco, Grand Island, NY, USA) containing 30% L929 cell culture supernatant for 5 days. The adherent cells were collected and seeded in 48- (2 × 10^5^ cells/well) or 6-well (2 × 10^6^ cells/well) plates. After overnight incubation at 37°C with 5% CO_2_, cells were infected with *M. abscessus* at an indicated multiplicity of infection (MOI) for various time points, and the culture supernatant was collected for cytokine measurement. For Western blotting, cellular proteins were extracted with a lysis buffer containing 1% Nonidet-P40 supplemented with a complete protease inhibitor cocktail (Roche, Mannheim, Germany) and 2 mM dithiothreitol.

### Inhibitor Assay

SB203580 and SP600125 were purchased as p38 and JNK MAPKs inhibitors, respectively, from Calbiochem (La Jolla, CA, USA). BMDMs were pretreated with 10 or 20 µM of the inhibitors for 2 h and then infected with *M. abscessus* at a MOI 1:10 (BMDMs:bacterium) for 24 h. The culture supernatant was then collected for cytokine and nitric oxide (NO) measurement.

### RT-PCR

mRNA was extracted from BMDMs using the TRI Reagent (Ambion, TX, USA) and complementary DNA was synthesized from 0.2 µg of mRNA using HelixCriptTM First-strand cDNA synthesis kit (NanoHelix, Korea). Quantitative real-time PCR was performed using the Qiagen SYBR green PCR kit (Hilden, Germany). PCR was performed using the Roter-GeneQ-Pure Detection system (QIAGEN). Actin was used for normalization. The following primers were used: iNOS forward: 5′-GGCAGCCTGTGAGACCTTTG-3′; iNOS reverse: 5′-GCATTGGAAGTGAAGCGTTTC-3′; Actin forward: 5′-CAACGAGCGGTTCCGATG-3′; Actin reverse: 5′-GCCACAGGATTCCATACCCA-3′.

### Nitrite Assay

Bone marrow-derived macrophages were infected with *M. abscessus* at a MOI 1:10 along with mouse IFN-γ (BioLegend, San Diego, CA, USA) in the absence or presence of 1 mM of N^G^-nitro-l-arginine methyl ester (l-NAME; Sigma Aldrich, St. Louis, MO, USA) for indicated times. Nitrite formation in culture supernatants was determined *via* the Griess reaction as described previously (19).

### Cytokine Measurement

The concentrations of IL-6, TNF-α, IL-1β, and IFN-γ in supernatants from BMDM cultures and lung tissue lysates were determined using a commercial ELISA kits (R&D Systems, Minneapolis, MN, USA).

### Western Blotting

Cellular lysates (25 μg/well) were separated using 10% SDS-PAGE and transferred to polyvinylidene fluoride membranes. Membranes were then incubated with primary antibodies to regular or phosphorylated IκB-α, p38, ERK, JNK (Cell signaling Technology, Beverly, MA, USA), and iNOS (BD Biosciences, San Jose, CA, USA). A primary antibody to β-actin (Santa Cruz Biotechnology, CA, USA) was used to verify equal loading of the protein samples. Following incubation with the relevant secondary antibodies, proteins were detected using an enhanced chemiluminescence reagent (Intron Biotechnology, Seongnam, Korea).

### Statistical Analysis

The statistical significance of differences between groups was determined by a two-tailed Student’s *t*-test or one-way ANOVA followed by the Bonferroni *post hoc* test for multigroup comparisons (GraphPad Prism 5; GraphPad Software Inc., La Jolla, CA, USA). Values of *P* that were less than 0.05 were considered statistically significant.

## Results

### The Absence of NOD2 Fails Bacterial Clearance and Exacerbates Pathology in the Lungs of *M. abscessus*-Infected Mice

**Figure 1:**
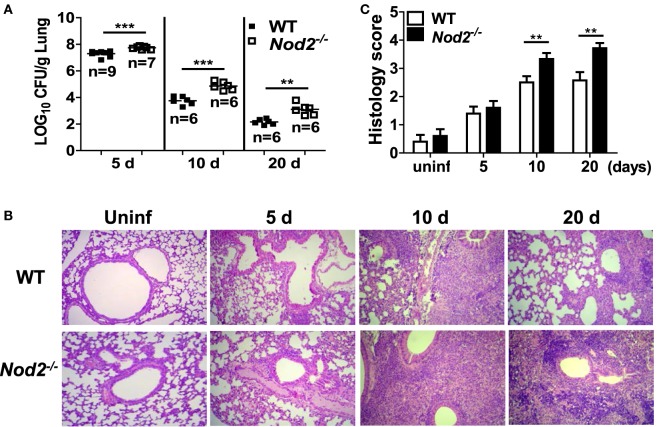
Nucleotide-binding oligomerization domain 2 (NOD2) controls bacterial growth and improves pathology in the lungs of mice with intranasal infection of *Mycobacterium abscessus*. **(A)** Wild-type (WT) and NOD2-deficient mice were i.n. infected with 1.5 × 10^7^ CFU of *M. abscessus* and the bacterial load in the lungs was determined at 5, 10, and 20 dpi. **(B,C)** The lung pathology was observed in H&E-stained sections and histology score in each group was determined as described in Section “Materials and Methods.” The results are from one representative experiment of two independent experiments (**P < 0.01, ***P < 0.001).

To determine whether NOD2 controls *in vivo* growth of *M. abscessus*, WT and NOD2-deficient mice were i.n. infected and the bacterial loads in the lungs were determined at 5, 10, and 20 dpi. No mortality of mice was observed in any group during the experiment. The bacterial loads in the lungs were significantly higher in NOD2-deficient mice as compared with those of the WT mice at all time points measured (Figure 1A). Upon histopathological examination, infiltration of inflammatory cells, mostly composed of monocytes and lymphocytes, were observed in the peribronchioles, and thickened alveolar septa were also seen in the lungs of *M. abscessus*-infected mice (Figure 1B). NOD2-deficient mice exhibited more severe lung pathology at 10 and 20 dpi (Figures 1B,C). Cellular composition in bronchoalveolar lavage fluids was not different between WT and NOD2-deficient mice groups at least until day 10 (data not shown). These results indicate that NOD2 may play an important role in host defense against *M. abscessus* pulmonary infection.

### NOD2 Is Essential for Cytokines Production in the Lungs of Mice Infected with *M. abscessus* during the Early Phase of Infection

**Figure 2:**
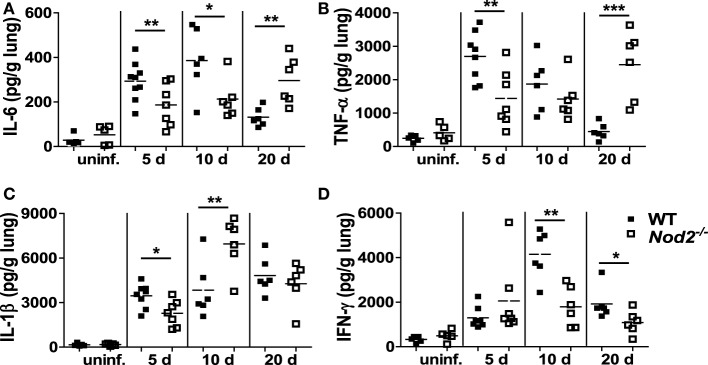
Nucleotide-binding oligomerization domain 2 (NOD2) is involved in optimal production of cytokines in the lungs of mice infected with *Mycobacterium abscessus* at early phase of infection (day 5). **(A–D)** Wild-type (WT) and NOD2-deficient mice were i.n. infected with *M. abscessus* and levels of IL-6, TNF-α, IL-1β, and IFN-γ in the lung homogenates were measured by ELISA. The number of animals used is shown in Figure 1A and that of uninfected group is five. The results are from one representative experiment of two independent experiments (*P < 0.05, **P < 0.01, ***P < 0.001).

We further investigated whether NOD2 deficiency influences cytokines production in the lungs of *M. abscessus*-infected mice. Levels of IL-6, TNF-α, and IL-1β were significantly lower in the lungs of NOD2-deficient mice as compared with those of WT mice at 5 dpi (Figures 2A–C). At 10 dpi, NOD2-deficient mice still produced less IL-6 compared to the WT mice, whereas TNF-α levels were comparable and IL-1β was increased in the lung homogenates of NOD2-deficient mice (Figures 2A–C). The levels of IL-6 and TNF-α were significantly higher in the lung homogenates of NOD2-deficient mice than those of the WT mice at 20 dpi (Figures 2A,B). When we measured levels of IFN-γ, the principal Th1 effector cytokine, NOD2-deficient mice exhibited less IFN-γ production at 10 and 20 dpi relative to that of the WT mice (Figure 2D). These results indicate that NOD2 is essential for the production of cytokines during the early stages of *M. abscessus* infection and may be required for optimal induction of the Th1-type immune response.

### Production of Cytokines and NO and Expression of iNOS Are Impaired in NOD2-Deficient Macrophages during *M. abscessus* Infection

**Figure 3:**
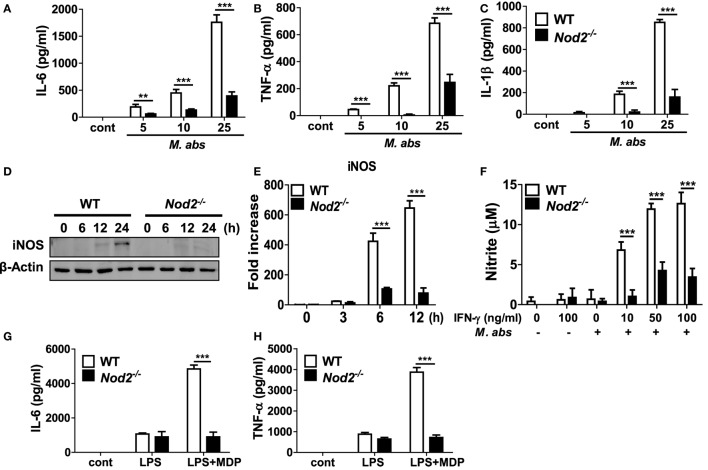
Production of cytokines and nitric oxide and expression of iNOS are impaired in nucleotide-binding oligomerization domain 2 (NOD2)-deficient macrophages in response to *Mycobacterium abscessus*. **(A–C)** Wild-type (WT) and NOD2-deficient bone marrow-derived macrophages (BMDMs) in triplicates were infected with indicated doses of *M. abscessus* for 12 h and levels of IL-6, TNF-α, and IL-1β in culture supernatants were measured by ELISA. **(D,E)** WT and NOD2-deficient BMDMs were infected with *M. abscessus* at a multiplicity of infection 1:10 and cellular proteins or mRNA were extracted at indicated times. The protein and gene expression of iNOS was determined by western blot and real-time PCR, respectively. **(F)** The cells were also infected with *M. abscessus* in the absence or presence of IFN-γ for 24 h and nitrite formation in culture supernatants was determined by Griess reaction. **(G,H)** IL-6 and TNF-α production in response to LPS (100 ng/ml) in the absence or presence of muramyl dipeptide (MDP) (10 µg/ml) was determined by ELISA to confirm the phenotype of NOD2-deficient BMDMs. **(A–H)** The results are from one representative experiment of three independent experiments (**P < 0.01, ***P < 0.001).

Next, we sought to determine the role of NOD2 in a cell-specific immune response using macrophages, one of the central cells of the innate immune system. Production of IL-6, TNF-α, and IL-1β increased in *M. abscessus*-infected BMDMs in a dose-dependent manner but which levels were significantly lower in NOD2-deficient cells (Figures 3A–C). We further compared iNOS expression and NO production by *M. abscessus* in WT and NOD2-deficient BMDMs. WT macrophages expressed iNOS protein in a time-dependent manner, reaching its maximal level of expression at the 24 h time point (Figure 3D). However, NOD2-deficient cells displayed minimal levels of iNOS expression throughout the time points (Figure 3D). Likewise, a high level of transcriptional upregulation of iNOS was observed in the WT macrophages infected with *M. abscessus* at 6 and 12 h after challenge, whereas the induction level was significantly lower in NOD2-deficient cells (Figure 3E). In addition, *M. abscessus* alone could not induce detectable levels of NO production in macrophages (Figure 3F). In the presence of IFN-γ, *M. abscessus*-induced substantial levels of NO production in macrophages, which was significantly less in NOD2-deficient cells (Figure 3F). To confirm the phenotype of NOD-deficient BMDMs, cytokines production in response to LPS was examined in the absence or presence of MDP. As expected, both WT and NOD2-deficient BMDMs produced comparable level of IL-6 and TNF-α in response to LPS (Figures 3G,H). LPS synergies with MDP to produce both cytokines in WT BMDMs, but not in NOD2-deficient cells (Figures 3G,H).

### P38 and JNK MAPKs Are Involved in NOD2-Mediated Production of Cytokines and NO in *M. abscessus*-Infected Macrophages

**Figure 4:**
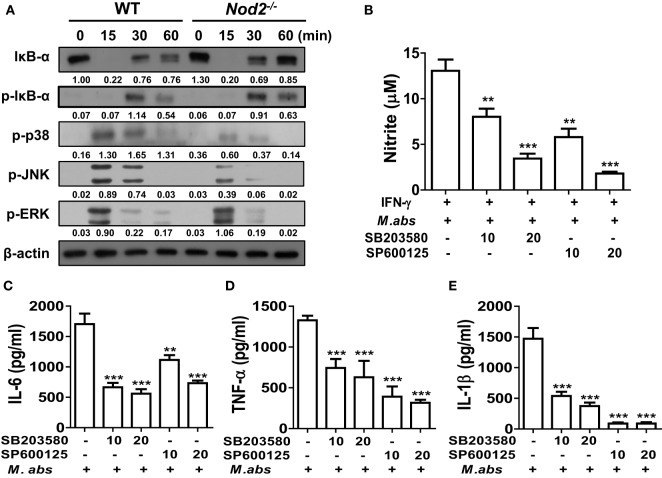
Nucleotide-binding oligomerization domain 2 (NOD2) mediates activation of p38 and JNK in macrophages in response to *Mycobacterium abscessus*, which contributes to nitric oxide production. **(A)** Bone marrow-derived macrophages (BMDMs) from wild-type (WT) and NOD2-deficient mice were infected with *M. abscessus* at a multiplicity of infection (MOI) 1:25 for indicated times and cellular proteins were extracted. Western blot analysis was performed to determine activation of NF-κB and mitogen-activated protein kinases (MAPKs) (p38, JNK, and ERK). β-Actin was used as a loading control. The quantification was performed by dividing the densities of each molecule by that of β-actin. Values are shown below the corresponding figures. **(B–E)** BMDMs were pretreated with SB203580 or SP600125 for 2 h and subsequently infected with *M. abscessus* at a MOI 1:10 for further 24 h. **(B)** IFN-γ (10 ng/ml) was co-treated to enhance nitrite formation. Nitrite concentration in culture supernatants was measured by Griess reaction. **(C–E)** IL-6, TNF-α, and IL-1β concentrations in culture supernatants were measured by ELISA. **(A–E)** The results are from one representative experiment of two independent experiments (**P < 0.01, ***P < 0.001).

We further investigated whether NOD2 is required for activations of NF-κB and MAPKs in BMDMs, the two key molecular factors regulating cytokine production in macrophages (20). Similar levels of IκB-α degradation and phosphorylation were detected at 15 and 30 min after *M. abscessus* infection between WT and NOD2-deficient cells (Figure 4A). At 15 min after the infection, phosphorylation of p38 and JNK was highly induced only in the WT BMDMs compared with those of NOD2-deficient cells (Figure 4A). Comparable levels of ERK phosphorylation were also detected in the WT and NOD2-deficient BMDMs at the 15 and 30 min time points, although the signal was undetectable in NOD2-deficient cells at 60 min (Figure 4A). An inhibitor assay revealed that both the p38 and JNK signaling pathways are involved in NO production in *M. abscessus*-infected BMDMs (Figure 4B). Moreover, *M. abscessus*-induced production of IL-6, TNF-α, and IL-1β was reduced in a dose-dependent manner by both inhibitors (Figures 4C–E). These findings suggest that *M. abscessus* activates p38 and JNK MAPKs *via* NOD2-mediated signaling pathway, which contributes to production of cytokines and NO in macrophages.

### NOD2-Mediated NO Production Is Essential for Restricting Intracellular Growth of *M. abscessus* within Macrophages

**Figure 5:**
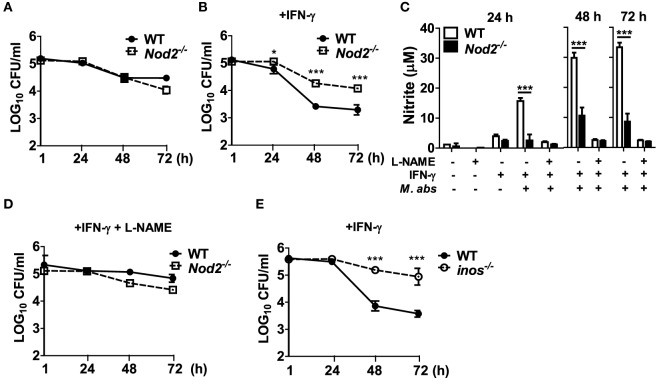
Nitric oxide is essential for nucleotide-binding oligomerization domain 2 (NOD2)-mediated restriction of intracellular survival of *Mycobacterium abscessus* in macrophages. **(A–D)** Wild-type (WT) and NOD2-deficient bone marrow-derived macrophages (BMDMs) were treated with *M. abscessus* [multiplicity of infection (MOI 1:10)] at the absence or presence of IFN-γ (10 ng/ml) and l-NAME (NOS inhibitor; 1 mM) (A, without IFN-γ and l-NAME; B, with IFN-γ; D, with IFN-γ and l-NAME). **(E)** Similarly, BMDMs from WT and iNOS-deficient mice were treated with *M. abscessus* (MOI 1:10) and IFN-γ. **(A,B,D,E)** The cell lysates were plated onto 7H10 agar at indicated times after infection and incubated for 5 days at 37°C and the number of bacteria was counted. **(C)** Nitrite concentration in culture supernatants was measured by Griess reaction. **(A–E)** The results are from one representative experiment of two independent experiments (*P < 0.05, ***P < 0.001).

Although there is no direct evidence showing the role of NO in controlling the growth of *M. abscessus*, it is well known that NO plays an important role in the host defense against microbial infections (21). As *M. abscessus*-induced NO production was impaired in NOD2-deficient macrophages (Figure 3F), we sought to determine whether NO contributes to restriction of the bacterial growth within macrophages. In the absence of IFN-γ treatment which is essential for NO production by *M. abscessus*, as shown in Figure 3F, there was no significant difference in intracellular growth of *M. abscessus* between WT and NOD-deficient BMDMs (Figure 5A). However, NOD2-deficient cells exhibited significantly higher CFU densities than did WT cells in the presence of IFN-γ at 24, 48, and 72 h after *M. abscessus* infection (Figure 5B). Moreover, treatment with l-NAME, a direct inhibitor of NOS, abolished *M. abscessus*-induced NO production (Figure 5C) and the difference in the intracellular bacterial CFUs between the WT and NOD2-deficient BMDMs (Figure 5D). Consistently, the ability to restrict the bacterial growth was impaired in iNOS-deficient BMDMs (Figure 5E). Taken together, NOD2 seems to be involved in the inhibition of intracellular survival and replication of *M. abscessus* by mediating iNOS expression and NO production in macrophages.

### Intranasal Administration of MDP Has a Protective Effect in Mice against *M. abscessus*

**Figure 6:**
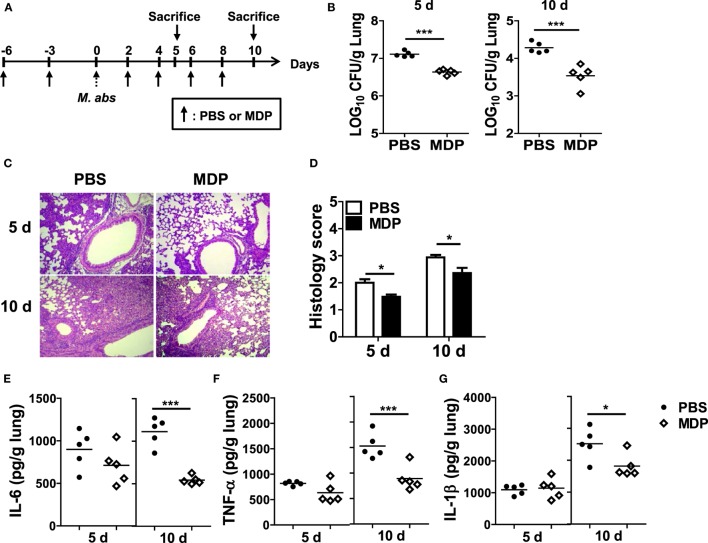
Intranasal administration of MDP promotes bacterial clearance and reduces lung pathology in *Mycobacterium abscessus*-infected mice. **(A)** Mice were i.n. administered with MDP [150 μg/animal in 30 µl phosphate buffered saline (PBS)] and infected with 1.5 × 10^7^ CFU of *M. abscessus* as scheduled in **(A)**. **(B)** The mice (*n* = 5 in each group) were sacrificed at 5 and 10 dpi and bacterial CFUs in the lungs were determined. **(C,D)** The lung pathology was observed in H&E-stained sections, and histology score in each group was determined. **(E–G)** Level of IL-6, TNF-α, and IL-1β in the lung homogenates was measured by ELISA (*P < 0.05, ***P < 0.001).

To determine whether NOD2 activation by MDP has a beneficial effect on the host defense against *M. abscessus* infection, MDP was given i.n. before and after *M. abscessus* infection as shown in Figure 6A. The bacterial loads in the lungs were significantly reduced in mice with MDP treatment at both 5 and 10 dpi versus the PBS-treated mice (Figure 6B). Paralleling the microbiological outcomes, the lung pathology was also improved in MDP-treated mice (Figures 6C,D). The MDP-treated mice also exhibited significantly lower levels of IL-6, TNF-α, and IL-1β in the lung homogenates than those of PBS-treated mice at day 10, although only marginal differences in the cytokine levels were observed between the two groups at day 5 (Figures 6E–G). These results suggest that NOD2-targeting molecules such as MDP may serve as preventive or therapeutic measures against *M. abscessus* infection.

## Discussion

In the present study, we revealed that NOD2 is essential for the optimal clearance of *M. abscessus* and reduced pathology in murine lungs. The bacterial load in the lungs was higher in NOD2-deficient mice even at 5 dpi, suggesting that NOD2 may contribute to inducing the innate immune response against *M. abscessus* during the early phase of infection. In line with these data, IL-6, TNF-α, and IL-1β levels in the lungs were significantly lower in NOD2-deficient mice at 5 dpi. Moreover, the *in vitro* study showed that the production of IL-6, TNF-α, and IL-1β in response to *M. abscessus* was impaired in NOD2-deficient macrophages. Such cytokines have been shown to be important in the host defense against *M. tuberculosis* infection (22–25). Besides pulmonary infections, TNF-α was also essential for bacterial clearance in the spleen and liver of mice infected by intravenous injection of *M. abscessus*, reducing the lethality of the infection (26). Taken together, NOD2 seems to inhibit *M. abscessus* infection by initiating cytokine production during the early phase of infection. However, during the late phase of infection, it is likely that NOD2 controls the growth of *M. abscessus* through different mechanisms, because despite levels of IL-6, TNF-α, and IL-1β being significantly higher in NOD2-deficient mice at 10 or 20 dpi, the bacterial burden was still higher in those mice. It is, therefore, assumed that increased bacterial densities induced high-level production of cytokines through a NOD2-independent pathway, which resulted in severe lung pathology inthose *M. abscessus*-infected mice. In addition, levels of IFN-γ were lower in the lungs of NOD2-deficient mice at 10 and 20 dpi. In previous publications, it has been reported that bacterial clearance in organs was impaired in IFN-γ- or RAG2-deficient mice infected with *M. abscessus* (26, 27). Moreover, activation of the antigen-specific T cell response was reduced in NOD2-deficient mice during mycobacterial infections (15). Although a more detailed role of NOD2 in Th1 immunity should be investigated in the future, our data indicate that NOD2 may be involved in the control of *M. abscessus* growth during the late phase of infection by regulating T cell response and IFN-γ-mediated signaling.

Macrophages are the representative innate immune cells and play an important role in host immunity against mycobacterial infections by producing cytokines and NO. In the present study, we demonstrated that NOD2 is essential for *M. abscessus*-induced cytokine production in murine macrophages. Although *N*-acetyl MDP exists in the peptidoglycan layer of most bacteria, mycobacteria and related actinomycetes possess *N*-acetyl muramic acid hydroxylase, which converts their MDP to an N-glycolylated form. *N*-glycolyl MDP is more potent than *N*-acetyl MDP in producing proinflammatory cytokines and activating NF-κB and JNK (17). Accordingly, it is assumed that NOD2 plays a much more important role in the host immunity against mycobacterial infections than it does in infections caused by other bacterial types. Indeed, a number of previous studies reported that a single deficiency of NOD2, even RIP2, does not influence cytokine production in murine macrophages in response to *Listeria, Yersinia, Pseudomonas*, and *E. coli* (20, 28–30). In contrast, production of cytokines in response to various *Mycobacterium* spp. was impaired in NOD2-deficient macrophages (31–34).

*Mycobacterium abscessus* could also activate NF-κB and all MAPKs (p38, ERK, and JNK) in murine BMDMs, but only p38 and JNK activation were influenced by NOD2 deficiency (Figure 4A). The inhibitor assay confirmed that both p38 and JNK signaling is necessary for optimal cytokine production in macrophages in response to *M. abscessus*. However, significance of p38 on *M. abscessus*-induced production of cytokines remains controversial. Shin et al. reported that *M. abscessus* induces p38 and ERK phosphorylation in murine macrophages (35). The inhibitor assay revealed that ERK, but not p38, is involved in TNF-α, IL-6, and IL-12p40 production by macrophages in response to *M. abscessus infection* (35). In contrast, Sampaio et al. reported that induction of TNF-α production in response to *M. abscessus* is abolished by the p38 inhibitor SB203580 in human monocytes (36). It is well known that pyridinyl imidazole, inhibitors of p38 MAPKs such as SB203580, inhibits autophosphorylation of RIP2 (37, 38), which is an adaptor protein for NOD1 and NOD2 (39). It is likely that SB203580 inhibits cytokines and NO production in *M. abscessus*-infected BMDMs by suppressing RIP2-mediated signaling. It should be further elucidated whether p38 directly regulates *M. abscessus*-induced production of cytokines and NO in macrophages using other experimental systems such as gene silencing or p38 knockout cells.

Nitric oxide plays an important role in the innate immune response and in the host defense against mycobacterial infection in mice, although there is still controversy about the role of NO in the control of *M. tuberculosis* in humans (21). iNOS-deficient mice displayed decreased survival times and impaired bacterial clearance in response to *M. tuberculosis* infection (40). NOD2 is required for iNOS expression and NO production in *M. tuberculosis*-infected macrophages (31, 41). Consistently, in the present study, transcriptional and translational expression of iNOS induced by *M. abscessus* was decreased in NOD2-deficient macrophages. Moreover, in the presence of IFN-γ, NOD2 deficiency led to impairment of NO production. NOD2 was also required for control of the bacterial growth in IFN-γ-treated cells, and l-NAME increased intracellular survival of the bacteria, suggesting that NOD2-mediated NO production may be crucial for restriction of *M. abscessus* growth within murine macrophages. In line with the NO production data, the bacterial growth control was impaired in iNOS-deficient macrophages. These findings were unique compared to previous reports studying *M. tuberculosis* infection. Although *M. tuberculosis*-induced NO production was impaired in NOD2-deficient macrophages, the bacterial replication was not different between WT and NOD2-deficient cells (31). It should be clarified whether or not NO-mediated restriction of bacterial replication in macrophages is specific to *M. abscessus* among the various *Mycobacterium* spp. In addition, in human macrophages, the role of NO in controlling the intracellular growth of *M. abscessus* may differ in that NOS activity is lower than in murine macrophages (42). Nevertheless, a recent study showed that compassionate inhaled NO treatment is very effective in bacterial clearance in cystic fibrosis patients with persistent *M. abscessus* infection (43). This implies that NO-based therapies can be effectively applied in patients with *M. abscessus* infection and support our results.

It has been known that MDP activation of NOD2 has protective effect against several diseases, such as colitis, atherosclerosis, and influenza infection, in mice (44–46). As NOD2 deficiency impairs bacterial clearance and pathology in the lungs of *M. abscessus*-infected mice, we sought to determine the effect of MDP-induced activation of NOD2 on host defense against the bacteria. Our results revealed that intranasal administration of MDP improves the bacterial clearance and pathology in the lungs of mice. As well as NOD2 activation, MDP can enhance TLRs-mediated immune responses in murine macrophages (20, 47). In addition, TLR2 contributes to early protective Th1 immune response against *M. abscessus* (16). Accordingly, NOD2-independent signaling such as TLR2 should not be overlooked in the protective effect of MDP, although MDP is well known as a NOD2-specific ligand (39). To clarify this, experiments using NOD2- or TLR2-deficient mice should be performed.

In summary, the present results suggest that (i) NOD2 deficiency leads to impaired bacterial clearance and thus more severe lung pathology in *M. abscessus*-infected mice, (ii) NOD2 contributes to the production of cytokines by regulating the activation of p38 and JNK in the infected macrophages, (iii) NOD2 is also involved in iNOS expression and NO production in *M. abscessus*-infected macrophages, which restricts intracellular replication of the bacteria, and (iv) *in vivo* administration of MDP enhances the host’s ability to control bacterial growth and reduces the severity of lung pathology in mice. In terms of that *M. abscessus* infection is dominant in patients with cystic fibrosis or immunosuppression, it seems to be important to know whether and how these underlying diseases modulate NOD2-mediated immune response in host. And if the results of this study are proved similar in humans, NOD2-mediated signaling can be a therapeutic target for *M. abscessus* infection.

## Ethics Statement

The animal studies were conducted under protocols approved by the Institutional Animal Care and the Use Committee of Chonnam National University (Approval No. CNU IACUC-YB-2015-32).

## Author Contributions

J-YL, S-JL, and E-JN performed experiments. D-JK, SS, and J-HP conceived and designed this study. M-SL contributed to analyze data. D-JK, S-JY, and J-HP wrote this manuscript.

## Conflict of Interest Statement

The authors declare that the research was conducted in the absence of any commercial or financial relationships that could be construed as a potential conflict of interest.
